# Comparison between Invasive Intervention and Conservative Treatment in Patients with In-Hospital Myocardial Infarctions: Results from the Regional Myocardial Infarction Registry of Saxony-Anhalt (RHESA) Study

**DOI:** 10.3390/jcm13082194

**Published:** 2024-04-10

**Authors:** Mohamad Assaf, Daniela Costa, Ljupcho Efremov, Karen Holland, Rafael Mikolajczyk

**Affiliations:** 1 Institute for Medical Epidemiology, Biometrics and Informatics, Martin Luther University Halle-Wittenberg, Magdeburger Str. 8, 06112 Halle (Saale), Germany; mohamad.assaf@uk-halle.de (M.A.); daniela.costa@uk-halle.de (D.C.); karen.holland@uk-halle.de (K.H.); 2 German Cancer Research Center (DKFZ), 69120 Heidelberg, Germany; ljupcho.efremov@dkfz-heidelberg.de

**Keywords:** in-hospital AMI, invasive intervention, conservative treatment, risk factors, 30-day mortality

## Abstract

**Background/Objectives**: In-hospital myocardial infarctions (AMIs) are less often treated with invasive intervention, compared to out-of-hospital AMIs. We aimed to identify the determinants of invasive intervention in patients with in-hospital AMIs and assess its association with mortality, compared to conservative treatment. **Methods**: This was a cross-sectional study of in-hospital AMIs in The Regional Myocardial Infarction Registry of Saxony-Anhalt. Patients’ characteristics and outcomes were compared based on the treatment strategy (invasive intervention vs. conservative treatment). Logistic regression was performed to assess the determinants of invasive intervention (vs. conservative treatment) and its association with 30-day mortality. **Results**: Nearly 67% of the patients (259/386) received invasive intervention, and the rest were treated conservatively. Those who were treated with an invasive intervention were younger and had a lower proportion of chronic heart failure than those treated conservatively. Age > 75 years compared to younger patients, pre-existing heart failure, and higher heart rate upon presentation were associated with lower odds of receiving invasive intervention. Hypertension (OR = 2.86, 95% CI [1.45–5.62]) and STEMI vs. NSTEMI (1.96, [1.10–3.68]) were associated with higher odds of invasive intervention. The adjusted odds of 30-day mortality were lower with invasive intervention compared to conservative treatment (0.25, [0.10–0.67]). **Conclusions**: One-third of the patients with in-hospital AMIs received conservative treatment. Younger age, absence of heart failure, lower heart rate, hypertension, and STEMI were determinants of invasive intervention usage. Invasive intervention had lower odds of 30-day mortality, but longitudinal studies are still needed to assess the efficacy of conservative vs. invasive strategies in in-hospital AMIs.

## 1. Introduction

Acute myocardial infractions (AMIs) occurring among patients hospitalized for other conditions, otherwise known as in-hospital AMIs, constitute between 1 and 11% of the total AMI cases managed in hospitals [1,2,3]. Compared to patients with out-of-hospital AMIs, those with in-hospital AMIs have a higher risk of in-hospital complications, as well as short- and long-term mortality [1,2,3,4,5,6,7,8,9]. Lower rates of invasive intervention (percutaneous coronary intervention and/or bypass surgery) in patients with in-hospital AMIs may be associated with the worse clinical outcomes observed in this group [1,4,8].

Insights on the determinants of invasive intervention compared to conservative treatment were provided by studies that solely included out-of-hospital AMIs or did not distinguish between out-of-hospital and in-hospital AMIs. These studies [10,11,12,13,14,15] identified patient-related determinants of invasive intervention utilization such as younger age, male sex, and lower heart rate on admission, in addition to the absence of specific pre-existing comorbidities such as stroke, chronic kidney disease, heart failure, and dementia. Moreover, a diagnosis of ST-segment elevation (STEMI) was found to be a determinant of invasive intervention usage compared to non-ST-segment myocardial infarction (NSTEMI) [10,12]. In contrast, less is known about the determinants of invasive intervention in patients with in-hospital AMIs specifically. Since in-hospital AMIs differ from their out-of-hospital counterpart in terms of associated risk factors, treatments, and consequently outcomes [1,2,3,4,5,6,7,8,9], it is important to identify the specific patterns of utilization and determinants of invasive intervention in patients with in-hospital AMIs.

Therefore, we aimed to examine the demographic, lifestyle, and clinical determinants of invasive intervention in patients with in-hospital AMIs, as well as its association with 30-day mortality. We compared the differences in the baseline characteristics of patients with in-hospital AMIs based on the treatment strategy (invasive vs. conservative) and identified factors associated with the use of invasive intervention. In addition, we assessed the 30-day mortality and post-AMI care of in-hospital AMI cases managed with an invasive vs. conservative strategy.

## 2. Methods

### 2.1. Study Design, Dataset Description, and Data Collection

In this cross-sectional study, we analyzed data from the population-based registry “The Regional Myocardial Infarction Registry of Saxony-Anhalt” (RHESA). The German federal state Saxony-Anhalt is known to have higher morbidity and mortality of AMIs compared to the other 15 federal states [16]. To identify contributing factors to the worse AMI outcomes and improve cardiovascular morbidity and mortality, the RHESA study was established in 2013. It includes fatal and non-fatal AMI cases occurring among patients older than 25 years, who reside in the urban region “Halle” or the rural region “Altmark” in Saxony-Anhalt [17,18].

Several rescue services centers, sixteen hospitals, and three public health departments participated in RHESA, enabling the identification of AMI cases. Trained physicians and nurses collected, via questionnaires, information related to the baseline characteristics of patients with AMIs, including pre-exiting chronic conditions and sociodemographic factors. In addition, they reviewed medical charts to obtain inpatient information related to the acute treatment of AMIs, complication occurrence, and discharge status of the included patients. Moreover, registration offices participating in RHESA informed the study personnel about the survival status of the included patients at different time points, to track mortality. Data collection was conducted through hospital collection forms (KEB). In the case of a non-fatal event or in-hospital death, a hospital physician or study assistant fills out the KEB based on medical chart review, in an anonymized/non-anonymized manner based on the availability of the consent form. In addition, an emergency protocol for consenting patients is submitted by the emergency doctors in ambulances, with information on symptom duration, arrival times, and emergency services provided during the transport. As for cases of pre-hospital deaths, the participating health departments share the death certificates with RHESA. In addition, those health departments send the last treating physicians or coroners a KEB questionnaire to fill out and submit back to RHESA. To determine the survival status for patients with AMIs who consented to participate in the registry, the registration offices are contacted across different time points. As for deceased patients, the corresponding health department would forward the death certificates to RHESA. Further follow-up studies using telephone interviews for patients participating in RHESA have been conducted since 2014, in order to obtain data about changes in cardiovascular risk factors and utilization of health services as well as cardiac rehabilitation.

In our current analysis, we included exclusively in-hospital AMI cases that were managed at the hospital, between 2013 and 2019. Patients with previous history of AMIs were excluded as only the first AMI occurrence was of interest. Additionally, we excluded patients with missing values for the availability of a cardiac catheterization laboratory where in-hospital myocardial infraction was initially diagnosed. In cases of transfer, patients with missing values for this variable in either the referring or the receiving hospitals were also excluded. This was conducted to ensure that the reason for opting for a conservative treatment was not the lack of a catheterization laboratory in the treating hospital. The characteristics and outcomes of the included patients are shown in Supplementary Table S1 of the Supplementary Materials.

### 2.2. Variables and Outcomes

AMIs were defined based on the Third Universal Definition of Myocardial Infarction [19], which describes AMIs as changes in the cardiac troponin by at least one unit above the 99th percentile of the upper reference associated with the presence of ischemic signs and/or symptoms. Information regarding the patients’ characteristics was obtained via questionnaires filled out by medical doctors or study nurses. This included age at AMI occurrence (in years, we then categorized into above or below 75 years), sex, region of residence (urban area of Halle and rural area of Altmark), height in meters and weight in Kilograms to calculate body mass index (BMI) that we categorized into four groups (<25, 25–29, 30–35, >35 kg/m^2^), smoking status (current, former, or never smoker), and comorbidities (presence of hyperlipidemia, hypertension, diabetes, chronic kidney disease, heart failure, atrial fibrillation, and/or history of stroke). In addition, the questionnaire included information on heart rate and systolic blood pressure upon presentation, electrocardiogram classification (STEMI vs. NSTEMI), treatments (Acetylsalicylic acid (ASS), P2Y12 inhibitor, heparin, thrombolytic drug, invasive intervention with either percutaneous coronary intervention (PCI), and/or bypass surgery), presence of shock upon presentation, onset of in-hospital complications, mortality, and time until death. The last section of the KEB questionnaire included a list of medications (ASS, P2Y12 receptor inhibitor, anticoagulant, ACE/ARB, beta-blocker, and statins), and the physicians or study nurses filling it out would choose the discharge medications for each participant based on the medical chart review. In-hospital complications were defined as having at least one or more of the following: intubation, another shock, re-infarct, stroke, severe bleed, or need for re-intervention. The availability of a cardiac catheterization laboratory in the hospital where the in-hospital AMI was first diagnosed was also collected. In the case of transfer to a second hospital, information on the availability of a catheterization laboratory in the second hospital was also obtained.

The main variable of interest was the treatment strategy: invasive intervention with PCI/bypass vs. no procedure (labeled as conservative treatment). The main outcome was 30-day mortality.

### 2.3. Statistical Analysis

Patients’ characteristics, risk factors, outcomes, and treatments were compared based on the treatment strategy (invasive intervention or conservative treatment). Categorical variables were reported as frequencies (percentages). The numerical variables age and heart rate were reported as the mean (standard deviation). The following variables had missing values greater than five percent: BMI, smoking status, information on comorbidities including chronic kidney disease, hyperlipidemia, hypertension, and chronic heart failure. Therefore, multiple imputation via the fully conditional specification method was used. Forty complete datasets were generated based on the rule that the number of imputations should be no less than the percentage of missing cases [20]. One logistic regression model was performed to identify the determinants of invasive intervention in patients with in-hospital AMIs. Then, a directed acyclic graph (DAG) was constructed using DAGitty software (Version 3.1) [21] (Supplementary Figure S1 in Supplementary Materials) in order to identify the minimum set of covariates to adjust for in the second logistic regression model assessing the association between treatment strategy and the outcome, 30-day mortality.

### 2.4. Description of Directed Acyclic Graph (DAG)

Since we have not found (to date) studies on the factors associated with the treatment strategy in in-hospital AMIs, we used the results of the first logistic regression model (dependent variable: invasive intervention) to draw associations between the exposure “treatment strategy” and the rest of the variables in a DAG. Associations between the outcome “30-day morality” and the other variables were based on a literature review. The variables included older age, sex, hypertension, hyperlipidemia, BMI, diabetes, atrial fibrillation, heart failure, chronic kidney disease, smoking status, history of stroke, heart rate on admission, and electrocardiogram (EKG) classification. The minimum set of variables to adjust for in the logistic regression analysis for the association between the treatment strategy and the dependent variable (30-day mortality) included age > 75 years, hypertension, heart failure, heart rate, and EKG classification. It is worth mentioning that Generalized Additive Model (GAM) analysis was conducted to investigate the potential non-linear relationship between the continuous variable heart rate and each of the two dependent variables, treatment strategy and 30-day mortality. The results revealed a linear association between heart rate and each of the dependent variables (not shown), thus warranting the retention of heart rate as a linear term in the final logistic regression models. The DAG revealed the following set of minimum variables to adjust for in the association between treatment strategy and 30-day mortality: age > 75 years, hypertension, heart failure, heart rate, and AMI classification (Supplementary Figure S1 in Supplementary Materials). These were entered as independent variables in the second logistic regression analysis to minimize bias in the association between treatment strategy and 30 d mortality.

We reported the odds ratios (ORs) and 95% confidence intervals (CIs) yielded by the regression models. In the sensitivity analysis, we repeated the regression analyses excluding patients who had an immediate death (within 24 h, *N* = 13), which we had kept in the initial models to mitigate immortal-time bias, in order to gain more insight into the robustness of our results. Finally, discharge medications for patients who survived beyond 30 days were compared based on the treatment strategy. All statistical analyses were conducted with R-Studio R ^®^ version 4.2.1 [22,23].

### 2.5. Ethical Consideration

RHESA was approved by the Ethics Committee of the Medical Faculty of the Martin Luther University Halle-Wittenberg (Nr.: 2020-188) and by the State Data Protection and Privacy Commissioner of Saxony-Anhalt. This study was conducted in accordance with the Declaration of Helsinki.

## 3. Results

**Figure 1:**
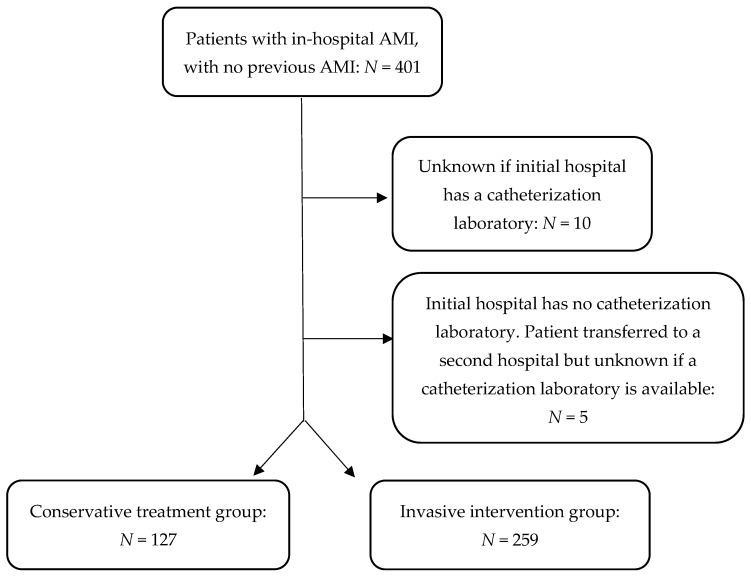
Total number of patients included in the analysis.

The total number of patients with in-hospital AMIs in our sample was 386 (Figure 1).

Nearly 67% of the patients underwent an invasive intervention while the rest received conservative treatments. The invasive intervention group was slightly younger than the conservative treatment group (70 ± 12 years [68.4–71.4] vs. 75 ± 12 years [73.2–77.6]). The proportion of females was slightly higher in the conservative group than in the invasive intervention group (51.1% [42.1–50] vs. 36.6% [30.6–42.3]). There were no differences in the proportions of pre-existing comorbidities between the two groups, with the exception of heart failure. A history of heart failure was less common among patients who were treated with an invasive intervention (25.9% [20.8–30.4] vs. 40.2% [31.9–48.8]). The proportion of patients from the rural area (Altmark) was higher in the conservative treatment group than in the invasive intervention group (59.1% [50.4–67.3] vs. 23.9% [19.1–29.4]) (Table 1).

Two-thirds of the patients in the conservative treatment group (*N* = 80) were first diagnosed with in-hospital AMIs in hospitals with no available cardiac catheterization laboratory. Of those, eleven died in the same hospital and seven were discharged alive without needing an intervention. The remaining 62 patients were transferred to hospitals with an available catheterization laboratory and still received no intervention. Among patients treated invasively, 30% (*N* = 78) were first diagnosed with in-hospital AMIs in hospitals without an available cardiac catheterization laboratory. All of them were transferred to a second hospital with a catheterization laboratory where they received the intervention. There was no difference in systolic blood pressure and heart rate between the two groups. STEMI was diagnosed more frequently in the invasive intervention group than in the conservative treatment group (27.8% [22.6–33.5] vs. 15.0% [9.6–21.2]). There was no difference in the proportion of heparin and thrombolytic medication administration between the two groups. Additionally, there were no major differences in terms of shock upon presentation and in-hospital complications. The outcome 30-day mortality was lower in the invasive intervention group (8.5% [5.6–12.4] vs. 18.9% [13.8–26.4]) (Table 2).

The multivariable logistic regression analysis for identifying the determinants of invasive intervention in patients with in-hospital AMIs revealed lower odds with increasing age among patients older than 75 years (adjusted OR = 0.85, 95% CI [0.76–0.94]), as well as with heart failure (adjusted OR = 0.52, 95% CI [0.30–0.90]) and increasing heart rate (adjusted OR = 0.98, 95% CI [0.97–0.99]). Hypertension, however, was associated with higher odds of receiving invasive intervention (OR = 2.86, 95% CI [1.45–5.62]). Compared to in-hospital NSTEMIs, patients with in-hospital STEMIs were 1.96 times more likely to receive invasive intervention (95% CI [1.10–3.68]). After adjusting for other factors, we found that there was no difference in the odds of utilization of invasive intervention between males and females (Table 3).

In terms of the main outcome, invasive intervention was associated with lower odds of 30-day mortality in comparison with conservative treatment (OR = 0.25, 95% CI [0.10–0.67]), after adjusting for relevant confounders. The diagnosis of in-hospital AMIs in hospitals with a catheterization laboratory was associated with higher odds of 30-day mortality compared to hospitals with unavailable catheterization laboratories and subsequent hospital transfer (8.75, 95% CI [2.68–25.39] (Table 4).

The region of residence showed no association with the outcome 30-day mortality.

Considering patients who survived beyond 30 days, the majority in both groups were discharged on ASS. The conservative treatment group had lower proportions of P2Y12 receptor inhibitor, beta-blocker, and statins medications upon discharge. Patients who were treated conservatively were more commonly discharged on an anticoagulant (37.9% [28.9–47.5] vs. 21.1% [16.3–26.6]) (Table 5).

In the sensitivity analysis, we repeated the regression analyses excluding 13 patients with in-hospital AMIs who died within the first 24 h. The determinants of invasive intervention remained the same, except that chronic kidney disease showed an association with the use of invasive intervention (Supplementary Table S2). The factors associated with 30-day mortality remained the same as in the former model (Supplementary Table S3).

## 4. Discussion

In line with previous findings from studies including only out-of-hospital AMIs, we found that the majority of the patients with in-hospital AMIs in our sample were treated with invasive intervention. Similar to what is known for out-of-hospital AMIs, invasive management of in-hospital AMI cases in our study was less common in patients with a higher age, heart failure, and NSTEMI. Our analysis revealed that lower age, hypertension, heart failure absence, lower heart rate, and STEMI were relevant determinants for utilizing invasive intervention in patients with in-hospital AMIs. In terms of outcomes, the 30-day mortality in the invasively managed group was lower than that in the conservatively treated group, whereby invasive intervention was associated with a 55% reduction in the odds of 30-day mortality.

Clinical trials and registry studies showed that invasive intervention is the favorable option for patients with stable coronary artery disease (CAD) [10,14,24] due to lower short- and long-term mortality. We also found that patients with in-hospital AMIs who are treated conservatively had higher odds of 30-day mortality, compared to those treated conservatively. However, a recent systematic review showed that there was no difference in post-AMI all-cause mortality between patients treated invasively and those treated conservatively [25], but the rate of major cardiac adverse events (MACEs) was lower with invasive intervention. Due to the low sample size of patients with complications in our study population, we could not confirm the association between the treatment strategy and occurrence of MACEs in the case of in-hospital AMIs. The European Society of Cardiology (ESC) provides guidelines for the acute and long-term management of AMIs. These guidelines are mostly based on studies that included only out-of-hospital AMIs or did not distinguish between in- and out-of-hospital AMIs. In fact, there is currently a lack of standardized protocols for the evaluation and risk stratification of patients with in-hospital AMIs [26]. According to the ESC, the mainstay treatment of STEMI is PCI, performed within 12 h of symptom onset. Coronary artery bypass graft (CABG) surgery may be utilized alternatively in AMI cases complicated by cardiogenic shock or mechanical complications [24,27]. As for non-ST-elevation entities of the acute coronary syndrome, the ESC supports the individual risk stratification of patients and the decision to accordingly opt for invasive intervention vs. conservative treatment [24]. The clarity of these guidelines regarding the default use of invasive intervention in STEMI compared to the need for a case-by-case assessment in NSTEMI could explain why STEMI patients in our sample were more likely to receive invasive intervention, relative to NSTEMI patients. Nonetheless, opting for no PCI in NSTEMI is considered a major risk factor for recurrent ischemic events. Thus, conservative treatment should be restricted to individual cases where the risks outweigh the benefits due to anatomical or clinical reasons. Such cases ought to be managed optimally with antiplatelet medications and secondary prevention, while taking into consideration the prevalent comorbidities [24]. To address the efficacy of conservative treatment vs. invasive intervention in elderly patients with NSTEMI, the British Heart Foundation is currently conducting a prospective and multicentric randomized controlled trial, the SENIOR-RITA Trial [28]. We anticipate that in this group as well, invasive intervention would be more favorable.

In terms of post-AMI management, the ESC recommends triple pharmacological treatment with an oral anticoagulant and dual antiplatelet therapy for the majority of STEMI patients [24,27]. We found that in-hospital AMI patients who were treated conservatively were less frequently discharged on P2Y12 receptor inhibitor, potentially due to an increased risk of bleeding, and especially that these patients were more commonly discharged on anticoagulants. In addition, physicians are recommended to discharge all AMI patients on statins, regardless of the cholesterol levels upon presentation, as this reduces the risk of early and long-term adverse cardiovascular events. The ESC additionally recommends the use of beta-blockers post-AMI (in cases of no contraindications), as well as ACE/ARB in AMI patients with hypertension, heart failure, and/or diabetes [24,27]. Contrarily to this, participants in our study who received conservative treatment were less frequently discharged on beta-blockers and statins, warranting the need for evaluation and optimization of the post-AMI care in this group.

Another interesting finding in our analysis was that patients who were staying in hospitals without a catheterization laboratory when they were initially diagnosed with an in-hospital AMI had lower odds of mortality than patients who were initially diagnosed with an in-hospital AMI in hospitals with available catheterization laboratories. We believe that patients who were initially present in more readily equipped hospitals were more likely sicker and comorbid than those who were present in hospitals not equipped with catheterization laboratories, contributing to higher mortality. One prospective study [29] involving patients with out-of-hospital AMIs showed that the availability of a PCI facility was the strongest predictor of the utilization of PCI as a treatment strategy. Patients who were directly admitted to hospitals readily equipped with PCI laboratories were five times (95% CI [3.6–7.9]) more likely to receive PCI, and this was independent of the time of symptom onset or hospital arrival. Another factor that could play a role in the choice of treatment strategy is the rationality. Evidence on the differences in the outcomes of AMIs between urban and rural regions remains conflicted. On one hand, patients from rural areas might have worse outcomes and higher mortality, potentially due to lower rates of invasive intervention in rural areas [30,31]. On the other hand, some studies found no association between rationality and the outcomes of AMIs [32,33]. Our results were in line with the latter. While the proportion of patients from the rural area was higher in the conservative treatment group, the multivariable analysis showed no association between the region of residence and 30-day mortality.

To understand why some patients with in-hospital AMIs receive conservative treatment despite the guidelines favoring invasive intervention, it is important to consider the various factors that play a role in the choice of the treatment strategy. Previous studies reported that older age and higher heart rate are negative determinants of invasive treatment in patients with AMIs [10,13,14]. We found that among patients with in-hospital AMIs, age (especially among those aged 75 years or older) and heart rate are implicated in the choice of treatment strategy. A high heart rate could be an indicator of hemodynamic instability, which could prompt physicians to avoid the use of invasive intervention. Evidence on the association between sex and the choice of treatment strategy in patients with AMIs is inconclusive. Some studies reported that women diagnosed with acute myocardial infractions are less likely to receive an invasive intervention [10,29]. One study identified an interaction between age and sex, whereby females older than 75 years were found to be equally likely to receive an invasive intervention [34], while another multicenter study reported no influence of sex on the choice of treatment strategy [35]. In our study, we found differences in the crude percentages of females between the two treatment groups, but in the adjusted analysis, we found that sex had no impact on the utilization of invasive intervention in patients with in-hospital myocardial infarctions.

In addition, certain comorbidities (mentioned previously) are associated with the underutilization of invasive intervention [10,11,12,14,15]. Of these comorbidities, only hypertension and the absence of heart failure were relevant determinants of invasive intervention in our analysis of in-hospital AMIs. Interestingly, unlike one study that found that hypertension was associated with conservative treatment in out-of-hospital STEMIs [11], we found that it is associated with higher odds of using invasive intervention in patients with in-hospital AMIs. Another potential determinant for not treating patients with in-hospital AMIs invasively may be multi-comorbidity rather than the presence or absence of specific comorbidities. This may be supported by the fact that the two treatment groups in our sample were homogenous in terms of the prevalence of pre-existing conditions. Negers et al. suggested that multi-comorbidity might be the most prominent clinical determinant in the physicians’ decision-making instead of the specific comorbidities, even though there is no clinical evidence to support the benefits of this practice [13]. Moreover, a longer time duration between symptom onset and treatment may be a contributing factor. The ESC recommends the use of conservative treatment as an alternative to invasive intervention in cases where the time from diagnosis of STEMI exceeds 2 h [27]. Given that in-hospital AMIs are more likely to have an atypical presentation and are associated with delayed diagnosis [7,8,9,26,36], conservative treatment would be a suitable alternative. It is also possible that the pathophysiological mechanisms contributing to in-hospital AMIs are related to a mismatch in oxygen demand/supply not arising from plaque rupture and thrombosis (Type 2 AMI), where invasive intervention would be of little benefit [37]. Finally, the acuity of the concomitant medical condition in patients with in-hospital AMIs might hinder the use of invasive intervention. Unfortunately, adjusting for the severity of the disease was not possible in our study, as this information was not available. It is interesting to point out that one would expect the unavailability of a catheterization laboratory to play a role in opting for a conservative treatment. However, this was not the case in our sample. We found that the majority of the patients in the conservative treatment group who were initially present in hospitals without catheterization laboratories were transferred to hospitals with a catheterization laboratory but still received no intervention.

The results of our study must be interpreted with caution, due to some limitations. The RHESA covers only two regions in the federal state of Saxony-Anhalt, and mortality and morbidity might vary across the different rural districts in Saxony-Anhalt. This limits the generalizability of our findings. Additionally, the 16 hospitals participating in the registry may differ in terms of the volume and availability of tertiary cardiac health services compared to those not participating. This could have an influence on the treatment decision. Finally, residual confounding could not be eliminated, since other relevant comorbidities, such as cancer and cognitive impairment, as well as the duration of symptoms and data on the use of coronary angiography and its findings, were not collected.

To the best of our knowledge, this is the first study assessing the determinants of invasive intervention in in-hospital AMIs. The substantial sample size represents the foremost strength of our research, given that in-hospital AMIs are not common. Furthermore, we conducted DAG analysis to identify the minimum set of variables sufficient to reduce bias in the multivariable analysis.

## 5. Conclusions

One in every three patients with an in-hospital AMI received a conservative treatment, but lower odds of 30-day mortality were observed with invasive intervention. Younger age, absence of heart failure, and STEMI were consistent determinants of invasive intervention use between out-of-hospital and in-hospital AMI cases. The remaining determinants of invasive intervention in out-of-hospital AMIs were not relevant in the case of in-hospital AMIs. Thus, the protocols informing the use of invasive intervention in out-of-hospital AMIs might not be translatable to in-hospital AMIs, prompting the potential need for adaptations of the guidelines. In addition, the long-term post-AMI pharmacological care in patients with in-hospital AMI cases is suboptimal, warranting further examination. Longitudinal studies are required to assess the efficacy of conservative treatment and its long-term effects in patients with in-hospital AMIs, compared to invasive intervention.

## Figures and Tables

**Table 1:** Distribution of patients’ characteristics and risk factors based on treatment strategy.

Total Number of In-Hospital AMIs *N* = 386	Conservative Treatment: *N* = 127		Invasive Intervention: *N* = 259	
	*N* (%) or Mean (SD)	95% CI	*N* (%) or Mean (SD)	95% CI
Characteristics				
Age (years)	75.4 (12.7)	73.2–77.6	69.9 (12.4)	68.4–71.4
Age > 75 years	84 (66.1)	57.6–73.9	111 (42.9)	36.9–48.9
Female	65 (51.1)	42.1–60	95 (36.6)	30.6–42.3
Region:				
Halle (urban)	52 (40.9)	32.7–54.3	197 (76.1)	70.6–80.9
Altmark (rural)	75 (59.1)	50.4–67.3	62 (23.9)	19.1–29.4
Body mass index group (kg/m^2^)				
<25	21 (16.5)	10.9–23.7	46 (17.8)	13.5–22.8
25–<30	63 (49.6)	41.0–58.2	135 (52.1)	46.0–58.2
30–35	30 (23.6)	16.9–31.5	61 (23.6)	18.7–29.0
>35	12 (9.4)	5.3–15.4	17 (6.6)	4.0–10.1
Smoking Status				
Never smoker	85 (66.9)	58.4–74.7	133 (51.4)	45.3–57.4
Smoker	28 (22.0)	15.5–29.8	89 (34.4)	28.8–40.3
Former smoker	14 (11.0)	6.5–17.3	37 (14.3)	10.4–18.9
Pre-existing comorbidities				
Diabetes	53 (41.7)	33.4–50.4	106 (40.9)	35.1–47.0
Hypertension	100 (78.7)	71.0–85.2	222 (85.7)	81.1–89.6
Hyperlipidemia	46 (36.2)	28.2–44.8	94 (36.6)	30.6–42.3
Stroke	20 (15.7)	10.2–22.8	26 (10.1)	6.8–14.1
Atrial fibrillation	35 (27.6)	20.4–35.8	66 (25.5)	20.5–31.0
Heart failure	51 (40.2)	31.9–48.8	67 (25.9)	20.8–30.4
Chronic kidney disease	52 (40.9)	32.7–49.6	91 (35.1)	29.5–41.1

Numerical variables are presented in the form of the mean (standard deviation); 95% CI of the mean. Categorical variables are shown in the form of frequency (%); 95% CI of the percentage. SD: standard deviation. CI: confidence interval.

**Table 2:** Distribution of inpatient clinical metrics, medical treatments, and outcomes of patients with in-hospital myocardial infarctions based on treatment strategy.

Total Number of In-Hospital AMIs: *N* = 386	Conservative Treatment: *N* = 127		Invasive Intervention: *N* = 259	
	*N* (%) or Mean (SD)	95% CI	*N* (%)	95% CI
Inpatient clinical metrics				
Patient initially found in a hospital with no cardiac catheter laboratory	80 (63)	54.4–71	78 (30.1)	24.8–35.9
If yes, number of patients transferred to a hospital with cardiac catheterization laboratory	11 died in original hospital (5 within 24 h)		0 died in KH1	
	7 were discharged home		0 were discharged home	
	62 were transferred to a hospital with a catheterization laboratory but still received no intervention		78 were transferred to a hospital with a catheterization laboratory where they received intervention	
Heart rate on presentation (beats/min)	88 (23)	84–93	82 (23)	79–86
Systolic blood pressure on presentation (mmHg)	140 (31)	135–146	139 (30)	136–143
STEMI	19 (15.0)	9.6–21.2	72 (27.8)	22.6–33.5
Occurrence of shock upon presentation	7 (5.5)	2.5–10.5	25 (9.7)	6.5–13.7
Initial medical treatments				
ASS	94 (74.0)	65.9–81.0	164 (63.3)	57.3–69.0
P2Y12 inhibitor	51 (40.2)	31.9–48.8	101 (39.0)	33.2–45.0
Heparin	80 (62.4)	53.0–69.4	180 (69.8)	63.7–75.2
Thrombolytic agent	1 (0.8)	0.1–3.6	7 (2.7)	1.2–5.2
Outcomes				
In-hospital complications	40 (31.5)	23.9–39.9	73 (28.2)	23.0–33.9
30-day mortality	24 (18.9)	13.8–26.4	22 (8.5)	5.6–12.4

Numerical variables are presented in the form of the mean (standard deviation); 95% CI of the mean. Categorical variables are shown in the form of frequency (%); 95% CI of the percentage. SD: standard deviation. CI: confidence interval. STEMI: ST-segment elevation myocardial infarction. ASS: Acetylsalicylic acid.

**Table 3:** Determinants of invasive intervention in patients with in-hospital myocardial infarctions, compared to conservative treatment.

Factors	Adjusted OR	95% CI
Age ≤ 75 years	0.99	0.95–1.03
Age > 75 years	0.85	0.76–0.94
Sex (reference: male)	1.16	0.63–2.12
BMI group (reference: <25 kg/m^2^)		
25–<30	1.54	0.71–3.36
30–35	1.02	0.42–2.44
>35	0.43	0.14–1.33
Smoking status (never smoker)		
Current smoker	1.41	0.66–2.99
Previous smoker	0.97	0.42–2.29
Diabetes	1.34	0.71–2.53
Hypertension	2.86	1.45–5.62
Hyperlipidemia	0.59	0.32–1.12
History of stroke	0.71	0.30–1.69
Atrial fibrillation	1.19	0.63–2.36
Chronic kidney disease	1.95	0.97–3.91
Heart failure	0.52	0.30–0.90
Heart rate	0.98	0.97–0.99
Systolic blood pressure	0.99	0.98–1.01
STEMI (reference: NSTEMI)	1.96	1.10–3.68

Variables included in the model: age (years), sex (reference: male), BMI group (reference: <25 kg/m^2^), smoking status (reference: never smoker), diabetes, hypertension, hyperlipidemia, history of stroke, atrial fibrillation, chronic kidney disease, heart failure, heart rate on admission, systolic blood pressure on admission, and STEMI classification (reference: NSTEMI). OR: odds ratio. CI: confidence interval. BMI: body mass index. STEMI: ST-segment elevation myocardial infarction. NSTEMI: non-ST-segment elevation myocardial infarction.

**Table 4:** Factors associated with 30-day mortality in patients with in-hospital myocardial infarctions.

Factors	Adjusted OR	95% CI
Invasive intervention	0.25	0.10–0.67
Available catheterization laboratory in the hospital where in-hospital AMI was diagnosed (reference: no)	8.75	2.68–25.39
Urban region (reference: rural)	0.22	0.06–1.20
Age ≤ 75 years	0.28	0.14–3.01
Age > 75 years	4.60	0.32–6.7
Hypertension	0.53	0.23–1.37
Heart failure	1.91	0.84–4.40
Heart rate upon admission	1.01	0.99–1.02
STEMI (reference: NSTEMI)	2.85	1.19–6.84

Variables included in the model: age (years), hypertension, heart failure, heart rate on admission, and STEMI classification (reference: NSTEMI). OR: odds ratio. CI: confidence interval. STEMI: ST-segment elevation myocardial infarction. NSTEMI: non-ST-segment elevation myocardial infarction.

**Table 5:** Comparison of discharge medications of patients who survived beyond 30 days, based on treatment strategy.

Number of Patients Who Survived beyond 30 Days after In-Hospital AMI Onset: *N* = 340	Conservative Treatment: *N* = 103		Invasive Intervention: *N* = 237	
	*N* (%)	95% CI	*N* (%)	95% CI
ASS	87 (84.5)	76.6–90.5	216 (91.1)	87.0–94.3
P2Y12 receptor inhibitor	52 (50.5)	40.9–60.0	192 (81.0)	75.7–85.6
Anticoagulant	39 (37.9)	28.9–47.5	50 (21.1)	16.3–26.6
ACE/ARB	68 (66.0)	56.5–74.6	173 (73.0)	67.1–78.3
Beta-blocker	72 (69.9)	60.6–78.1	204 (86.1)	81.2–90.0
Statin	47 (45.6)	36.2–55.3	179 (75.5)	69.8–80.7

Variables are shown in the form of frequency (%); 95% CI of the percentage. ASS: Acetylsalicylic acid. ACE/ARB: Angiotensin converting enzyme inhibitors and/or Angiotensin II receptor blocker.

## Data Availability

The datasets used and/or analyzed during the current study are available from the corresponding author on request.

## Supplementary Materials

The following are available online at https://www.mdpi.com/article/10.3390/jcm13082194/s1, Table S1: Characteristics and outcomes of patients with in-hospital myocardial infarctions. Table S2: Results of the sensitivity analysis after removing those who died immediately (*N* = 13) showing determinants of invasive intervention in patients with in-hospital myocardial infarctions, compared to conservative treatment. Table S3: Results of the sensitivity analysis after removing those who died immediately (*N* = 13) showing factors associated with 30-day mortality in patients with in-hospital myocardial infarction. Figure S1: Directed acyclic graph (DAG) showing the hypothesized association between invasive intervention (exposure) and 30-day mortality (outcome). Description of variable selection in the directed acyclic analysis (DAG): Variables associated with the treatment strategy were identified based on the results of the first logistic regression analysis (dependent variable: invasive intervention and reference conservative treatment), due to the lack of previous studies on predictors of invasive intervention in in-hospital AMI, compared to conservative treatment. The identified factors included: older age (>75 years), heart rate, hypertension, heart failure, chronic kidney disease (CKD) and Electrocardiogram (EKG) classification. Variables associated with the outcome 30-day mortality based on literature review included: older age, sex, hypertension, hyperlipidemia, BMI, diabetes, Atrial fibrillation, heart failure, CKD, smoking status, history of stroke, heart rate on admission and EKG classification. The minimum set of variables to adjust for in the logistic regression analysis for the association between treatment strategy and the main outcome (30-day mortality) included: age > 75 years, hypertension, heart failure, heart rate and EKG classification. It is worth mentioning that Generalized Additive Model (GAM) analysis was conducted to investigate the potential non-linear relationship between the continuous variable heart rate and each of the two dependent variables; treatment strategy and 30-day mortality. The results revealed a linear association between heart rate and each of the dependent variables (not shown), thus warranting the retention of heart rate as a linear term in the final logistic regression models. CKD: chronic kidney disease; A fib: atrial fibrillation; EKG: electrocardiogram.

## Abbreviations

AMI	Acute myocardial infarction
BMI	Body mass index
CI	Confidence interval
RHESA	The Regional Myocardial Infarction Registry of Saxony-Anhalt
NSTEMI	Non-ST segment elevation myocardial infarction
SD	Standard deviation
STEMI	ST-segment elevation myocardial infarction
OHMI	Out-of-hospital myocardial infarction
OR	Odds ratio
ESC	European Society of Cardiology
CABG	Coronary artery bypass surgery
PCI	Percutaneous coronary intervention
ACE/ARB	Angiotensin converting enzyme inhibitors and/or Angiotensin II receptor blocker
DAG	Directed acyclic graph
CAD	Coronary artery disease
