# Supplementary material for: Comparison between Invasive Intervention and Conservative Treatment in Patients with In-Hospital Myocardial Infarctions: Results from the Regional Myocardial Infarction Registry of Saxony-Anhalt (RHESA) Study
Source: J Clin Med. 2024 Apr 10;13(8):2194. doi: 10.3390/jcm13082194 (PMC11050707; doi:10.3390/jcm13082194)
Supplement: Supplementary file 1 [file jcm-13-02194-s001.zip › jcm-2921069-supplementary.pdf]

Journal of
Clinical Medicine

MDPI

Supplementary Materials

Table S1. Characteristics and outcomes of patients with in-hospital myocardial infarctions.

Total = 401	Frequency (%) or mean (SD)
Age (years)	71.76 (75.1)
> 75 years	204 (50.9)
Female	160 (39.9)
Altmark (Rural region)	146 (36.4)
Halle (Urban region)	255 (63.6)
Body mass index group (kg/m²)	
<25	71 (17.7)
25 - <30	203 (50.6)
30-35	96 (23.9)
>35	30 (7.5)
Diabetes	164 (40.9)
Hypertension	334 (83.3)
Hyperlipidemia	144 (35.9)
History of stroke	48 (12.0)
Atrial fibrillation	108 (26.9)
History of heart failure	123 (30.7)
Chronic kidney disease	149 (37.2)
None smoker	227 (56.6)
Smoker	120 (29.9)
Former smoker	54 (13.5)
NSTEMI	299 (74.6)
STEMI	93 (23.2)
Invasive intervention	260 (64.8)
Conservative treatment	141 (35.2)
PCI	240 (59.9)
Average time to PCI (hours)	9 (6)
Bypass surgery	24 (6.0)
Average time to surgery (days)	4 (6)
In-hospital complications	119 (29.7)
30-day mortality	52 (13.0)

Numerical variables are presented in the form mean (standard deviation), and categorical variables in the form frequency (%). SD: standard deviation. NSTEMI: Non-ST-segment myocardial infarction. STEMI: ST-segment elevation myocardial infarction. PCI: Percutaneous coronary intervention.

Table S2. Results of the sensitivity analysis after removing those who died immediately (N= 13) showing determinants of invasive intervention in patients with in-hospital myocardial infarctions, compared to conservative treatment.

Factors	Adjusted OR	95% CI
Age ≤ 75 years	0.98	0.95 – 1.02
Age > 75 years	0.85	0.76 – 0.94
Sex (reference: male)	0.92	0.51 – 1.60
BMI group (reference <25 kg/m²)		
25- <30	1.53	0.73 – 3.37
30-35	1.04	0.46 – 2.46
>35	0.45	0.17 – 1.30

Smoking status (never smoker)		
Current smoker	1.45	0.67 – 2.97
Previous smoker	0.96	0.40 – 2.29
Diabetes	1.38	0.74 – 2.57
Hypertension	3.25	1.51 – 7.03
Hyperlipidemia	0.56	0.30 – 1.04
History of stroke	0.72	0.30 – 1.73
Atrial fibrillation	1.20	0.61 – 2.35
Chronic kidney disease	2.00	1.02 – 3.94
Heart failure	0.62	0.32 – 0.88
Heart rate	0.98	0.97 – 0.99
Systolic blood pressure	0.99	0.98 – 1.01
STEMI (reference NSTEMI)	1.77	1.10 – 3.50

Variables included in the model: age (years), sex (reference: male), BMI group (reference: <25 kg/m2), smoking status (reference: never smoker), diabetes, hypertension, Hyperlipidemia, history of stroke, atrial fibrillation, chronic kidney disease, heart failure, heart rate on admission, systolic blood pressure on admission, STEMI classification (reference: NSTEMI). OR: odds ratio. CI: confidence interval. BMI: body mass index. STEMI: ST-segment elevation myocardial infarction. NSTEMI: Non-ST-segment elevation myocardial infarction.

Table S3. Results of the sensitivity analysis after removing those who died immediately (N= 13) shpwing factors associated with 30-day mortality in patients with in-hospital myocardial infarction.

Factors	Adjusted OR	95% CI
**Invasive intervention**	0.39	0.14- 0.88
**First hospital with catheter laboratory (reference: no)**	9.51	1.58 – 27.74
**Urban region (reference: rural)**	0.24	0.06-1.12
Age ≤ 75 years	0.67	0.34 – 1.50
Age >75 years	1.49	0.67 – 3.32
Hypertension	0.32	0.13- 0.98
Heart failure	1.73	0.67 – 4.49
Heart rate upon admission	1.01	0.98 – 1.02
STEMI (reference NSTEMI)	1.49	0.51 – 4.36

Variables included in the model: age (years), hypertension, heart failure, heart rate on admission, STEMI classification (reference: NSTEMI). OR: odds ratio. CI: confidence interval. STEMI: ST-segment elevation myocardial infarction. NSTEMI: Non-ST-segment elevation myocardial infarction.

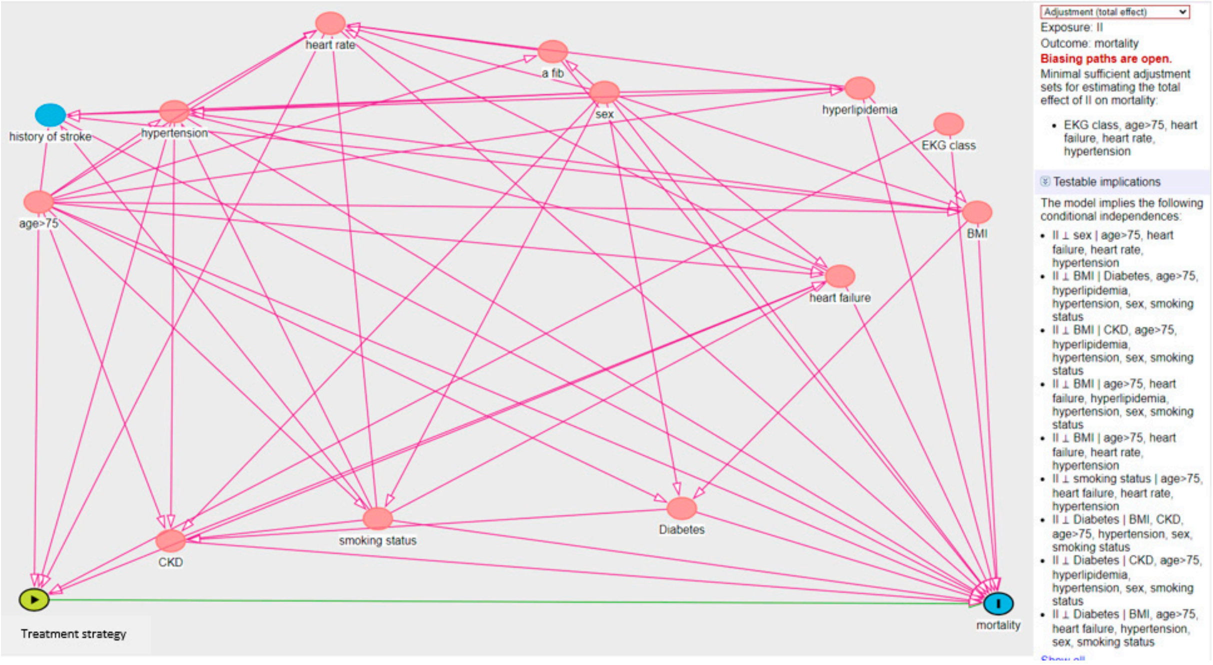

Figure S1. Directed acyclic graph (DAG) showing the hypothesized association between invasive intervention (exposure) and 30-day mortality (outcome). Description of variable selection in the directed acyclic analysis (DAG): Variables associated with the treatment strategy were identified based on the results of the first logistic regression analysis (dependent variable: invasive intervention and reference conservative treatment), due to the lack of previous studies on predictors of invasive intervention in in-hospital AMI, compared to conservative treatment. The identified factors included: older age (>75 years), heart rate, hypertension, heart failure, chronic kidney disease (CKD) and Electrocardiogram (EKG) classification. Variables associated with the outcome 30-day mortality based on literature review included: older age, sex, hypertension, hyperlipidemia, BMI, diabetes, Atrial fibrillation, heart failure, CKD, smoking status, history of stroke, heart rate on admission and EKG classification. The minimum set of variables to adjust for in the logistic regression analysis for the association between treatment strategy and the main outcome (30-day mortality) included: age > 75 years, hypertension, heart failure, heart rate and EKG classification. It is worth mentioning that Generalized Additive Model (GAM) analysis was conducted to investigate the potential non-linear relationship between the continuous variable heart rate and each of the two dependent variables; treatment strategy and 30-day mortality. The results revealed a linear association between heart rate and each of the dependent variables (not shown), thus warranting the retention of heart rate as a linear term in the final logistic regression models. CKD: chronic kidney disease; A fib: atrial fibrillation; EKG: electrocardiogram.
